# Differential expressions of FASN, SCD, and FABP4 genes in the ribeye muscle of omega-3 oil-supplemented Tattykeel Australian White lambs

**DOI:** 10.1186/s12864-023-09771-x

**Published:** 2023-11-06

**Authors:** John Roger Otto, Shedrach Benjamin Pewan, Richard Crawford Edmunds, Felista Waithira Mwangi, Robert Tumwesigye Kinobe, Oyelola Abdulwasiu Adegboye, Aduli Enoch Othniel Malau-Aduli

**Affiliations:** 1 School of Environmental and Life Sciences, College of Engineering, Science and Environment, The University of Newcastle, Callaghan, NSW 2308 Australia; 2 National Veterinary Research Institute, Private Mail Bag 01, Vom, Plateau State Nigeria; 3 Australian Institute of Marine Science, Townsville, QLD 4810 Australia; 4 School of Medicine and Public Health, College of Health, Medicine and Wellbeing, The University of Newcastle, Callaghan, NSW 2308 Australia; 5 College of Public Health, Medical and Veterinary Sciences, James Cook University, Townsville, QLD 4811 Australia; 6 Menzies School of Health Research, Charles Darwin University, Casuarina, NT 0811 Australia

**Keywords:** Tattykeel Australian White, Fatty acid binding protein 4, Fatty acid synthase, Stearoyl-CoA desaturase, Omega-3 fortified diets, Unsaturated fatty acids, Ribeye muscle

## Abstract

**Background:**

The concept of the functional nutritional value of health-beneficial omega-3 long-chain polyunsaturated fatty acids (n-3 LC-PUFA) is becoming a phenomenon among red meat consumers globally. This study examined the expressions of three lipogenic genes (fatty acid binding protein 4, *FABP4,* fatty acid synthase, *FASN*; and stearoyl-CoA desaturase, *SCD*) in the ribeye (*Longissimus thoracis et lumborum*) muscle of Tattykeel Australian White (TAW) lambs fed fortified omega-3 diets and correlations with fatty acids. To answer the research question, “are there differences in the expression of lipogenic genes between control, MSM whole grain and omega-3 supplemented lambs?”, we tested the hypothesis that fortification of lamb diets with omega-3 will lead to a down-regulation of lipogenic genes. Seventy-five six-month old TAW lambs were randomly allocated to the (1) omega-3 oil-fortified grain pellets, (2) unfortified grain pellets (control) or (3) unfortified MSM whole grain pellets diet supplements to generate three treatments of 25 lambs each. The feeding trial lasted 47 days.

**Results:**

From the Kruskal-Wallis test, the results showed a striking disparity in lipogenic gene expression between the three dietary treatments in which the *FABP4* gene was significantly up-regulated by 3-folds in the muscles of lambs fed MSM Milling (MSM) whole grain diet compared to the omega-3 and control diets. A negative correlation was observed between *FASN* gene expression and intramuscular fat (IMF), eicosapentaenoic acid (EPA), total polyunsaturated fatty acids (PUFA), omega-6 polyunsaturated fatty acids (n-6 PUFA) and monounsaturated fatty acids (MUFA). The *FABP4* gene expression was positively correlated (P < 0.05) with EPA and docosahexaenoic acid (DHA).

**Conclusion:**

Taken together, this study’s results suggest that *FABP4* and *FASN* genes perform an important role in the biosynthesis of fatty acids in the ribeye muscle of TAW lambs, and supplementary diet composition is an important factor influencing their expressions.

**Supplementary Information:**

The online version contains supplementary material available at 10.1186/s12864-023-09771-x.

## Background

The perception of a healthy nutrient-dense food is increasingly becoming a global and topical discourse amongst meat consumers due to knowledge advancements in medicine, science, and technology that have changed the lifestyles of the populace [1]. The fortification of livestock diets to increase the content of health-claimable fatty acids remains a viable strategy for improving meat quality because fatty acids are important building blocks for cellular structures, tissues, and organs [2]. They are also an integral part of the metabolic processes of synthesising and coordinating the roles of essential biologically active elements [3]. Long chain polyunsaturated fatty acids (LC-PUFA) are required for various biological and physiological processes, but mammals cannot synthesise them [4–6] because they lack Δ12 and Δ15 fatty acid desaturase enzymes, thus necessitating the need for dietary supplementation [6]. The α-linolenic (ALA) and linoleic (LA) acids are precursors of n-3 and n-6 LC-PUFA, respectively. ALA is converted to the more potent n-3 LC-PUFA, such as docosahexaenoic acid (DHA) and eicosapentaenoic acid (EPA) through the *de novo* synthesis metabolic pathway [7]. These fatty acids contribute to memory improvement, the elevation of visual acuity, the depression of high blood pressure [8], reduction of the risks of inflammatory and degenerative diseases such as cardiovascular diseases, cancer, skin conditions, metabolic syndrome, diabetic neuropathy, allergies, asthma, arthritis and the enhancement of immune function [7, 9, 10]. Similarly, LA is a precursor for the synthesis of arachidonic acid (ARA), an n-6 LC-PUFA that is converted to prostaglandins, leukotrienes, and other associated compounds. However, diets rich in n-6 PUFA are linked to inflammation, blood vessel constriction, and platelet aggregation [11, 12]. Therefore, a high intake of LA relative to ALA, has been reported to interfere with ALA desaturation and elongation pathways [13] as both LA and ALA utilise the same metabolic pathway for synthesising DHA and EPA [14].

An animal’s diet influences its meat fatty acid composition [15], through absorption of the dietary fatty acids [16] and modulation of cellular pathways such as lipogenic gene expression patterns [17]. Gene expression analysis can shed some light on the transcriptional pathways involved in the synthesis of functional gene products. Thus, identifying the framework of gene expression is vital to unravelling the molecular mechanisms controlling complex traits [18]. Only a few studies have evaluated dietary regulation of lipogenic gene expression in the ovine muscles [19–21]. In addition, the reported effect of dietary omega-3 rich supplements on gene expression is inconsistent. For instance, Fan et al. [22] reported an increase in the SCD gene expression, while Deng et al. [23] reported a decline in the SCD gene expression in the *longissimus thoracis* of Hu sheep. Hence, to better comprehend the genetic regulation of fatty acid deposition in Tattykeel Australian White (TAW) lambs, the ribeye muscle from lambs on diverse dietary supplements was utilised to provide a more detailed lipogenic gene expression pattern. To our knowledge, there is no published literature on the impact of dietary supplementation on transcriptional lipogenic gene expression in TAW lambs, hence the objective of this study as the first of its kind, was to fill this gap. We hypothesised that dietary fortification with omega-3 oils influences the transcriptional expression of lipogenic genes in the ribeye muscle in TAW lambs.

## Results

**Fig. 1:**
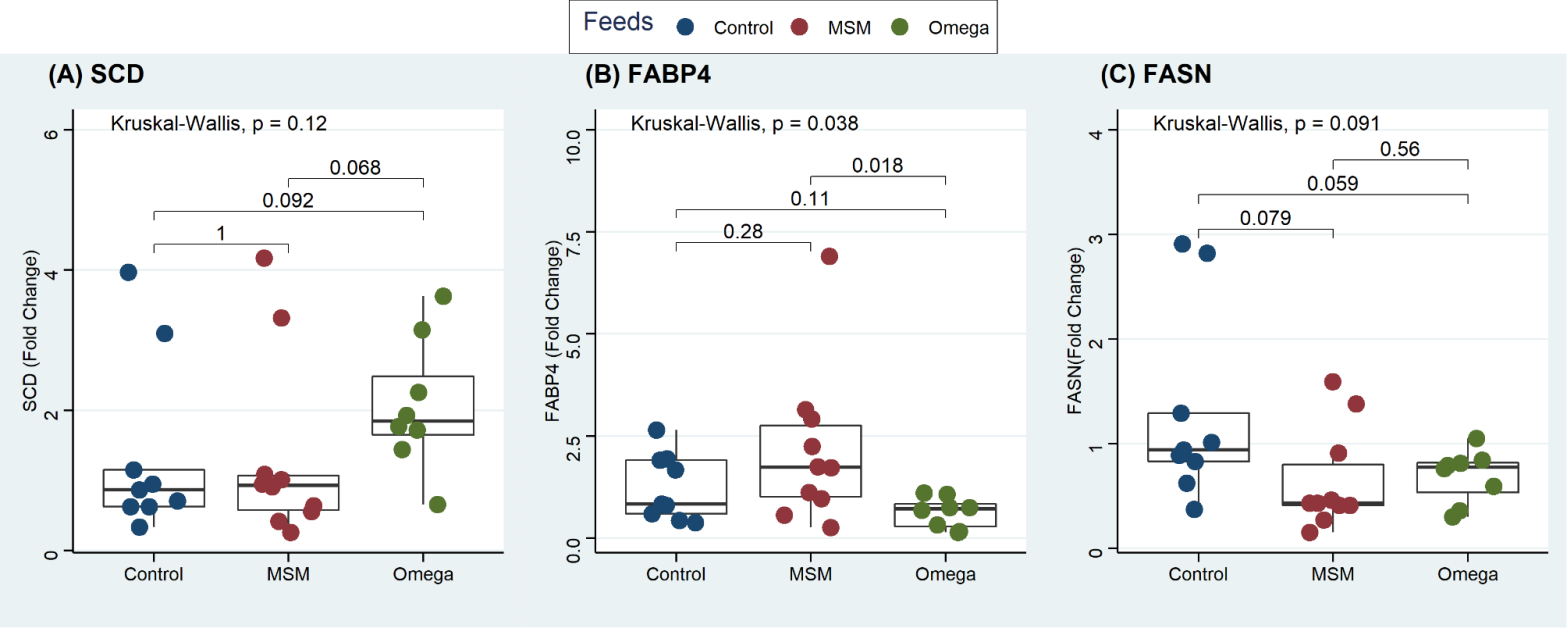
Observed fold-change differences in *SCD* (**A**), *FABP4* (**B**), and *FASN* (**C**) lipogenic gene expressions in the ribeye muscle tissue of lambs in the control, MSM whole grain and omega-3 dietary treatments

The three lipogenic genes showed marked variation in expression levels within the ribeye muscle of lambs in all three dietary supplementation groups (Fig. 1). The Kruskal-Wallis test showed that the *SCD* gene expression did not differ between treatments (Fig. 1A; P = 0.12). In Fig. 1B, the *FABP4* gene was up-regulated 3-folds in lambs fed the MSM whole grain diet compared to the omega-3 fortified diet (P = 0.018). However, the expression of *FABP4* between the control and the unfortified MSM whole grain or omega-3 fortified diet fed lambs did not differ (P ≥ 0.11). The expression of the *FASN* gene tended to be higher in the control lambs than in the MSM whole grain (P = 0.079) and the omega-3 fortified diet (P = 0.059). However, *FASN* gene expression between lambs fed MSM whole grain or the omega-3 fortified diet did not differ (P = 0.56).

**Fig. 2:**
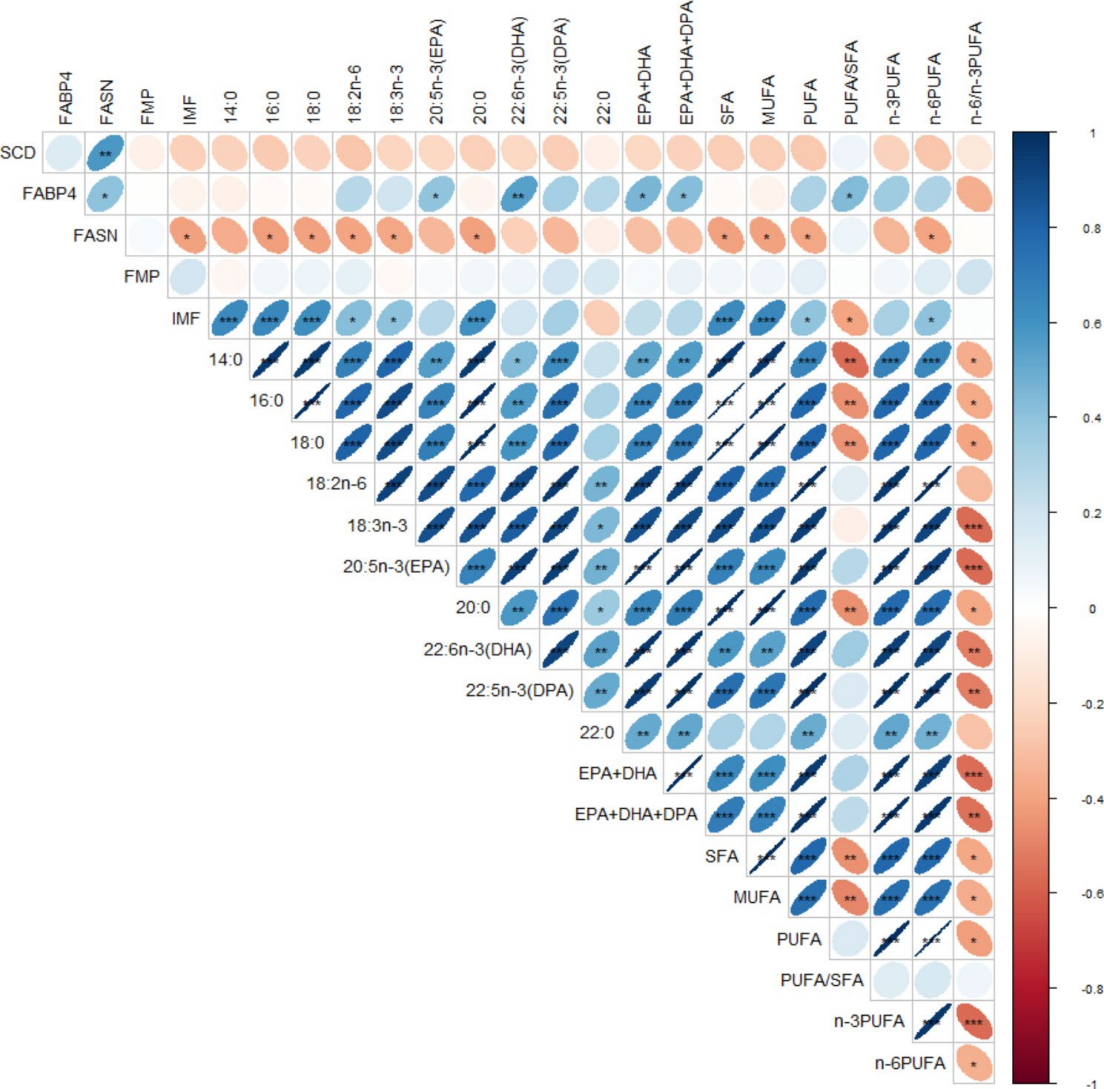
Expressions of *SCD*, *FASN* and *FABP4* genes and correlations with meat quality traits in the ribeye muscle of TAW lambs supplemented with MSM whole grain, omega-3 fortified or control diets (* < 0.05, ** < 0.01, *** < 0.001)

Spearman correlations between the fold changes in *SCD*, *FABP4*, and *FASN* gene expressions and meat quality traits measured are shown in Fig. 2. Some negative associations (P < 0.05) between IMF, total saturated fatty acids (SFA), individual SFA (C16:0, C18:0, C18:2n-6, C18:3n-3 and C20:0), monounsaturated fatty acids (MUFA), PUFA and n-6 PUFA, and fold changes in the expression of the *FASN* gene were observed. On the other hand, positive correlations (P < 0.05) were detected between the *FABP4* gene fold changes and DHA, EPA, EPA + DHA, EPA + DHA + DPA and PUFA/SFA ratio. However, the correlations between the *SCD* gene expression and meat quality traits did not attain statistical significance (P > 0.05).

The effects of *SCD*, *FABP4*, and *FASN* genes expression on meat quality traits in the ribeye muscle of TAW lambs supplemented with omega-3 fortified diets are depicted in Table 1. The results indicate that the effect of *SCD* gene expression on all the meat quality traits was negligible. The *FABP4* gene significantly influenced LA (C18:2n-6), ALA (C18:3n-3), EPA (C20:5n-3), DHA (C22:6n-3), DPA (C22:5n-3), EPA + DHA + DPA, n-3 PUFA and n-6 PUFA while the *FASN* gene expression influenced the IMF, C18:0, LA, EPA, C20:0, total PUFA and n-6 PUFA (P < 0.05).

**Table 1:** Effect of *SCD, FABP4, and FASN* gene expressions on meat quality traits in the ribeye muscle of supplemented TAW lambs

Dependent variable	*SCD*			*FABP4*			*FASN*		
	Est.	Lower	Upper	Est.	Lower	Upper	Est.	Lower	Upper
Fat Melting Point	0	-0.473	0.333	0	-0.528	0.686	0.262	-0.728	0.312
Intramuscular Fat	-0.184	-0.473	0.067	0.045	-0.567	0.2	**-0.586**	**-1.443**	**-0.367**
C14:0	-0.538	-4.761	0.679	-0.297	-3.261	2.732	-5.184	-16.185	0.065
C16:0	6.327	-67.754	12.902	-5.951	-18.975	27.225	-65.862	-157.254	14.556
C18:0	3.982	-36.763	12.405	-2.009	-15.966	19.41	**-37.923**	**-104.016**	**-0.077**
C18:2n-6	-2.903	-16.197	5.597	**8.657**	**6.214**	**13.435**	**-17.773**	**-50.658**	**-0.104**
C18:3n-3	0.13	-2.435	0.282	**1.057**	**0.293**	**2.314**	-2.839	-8.764	0.322
C20:5n-3	-0.708	-1.757	0.617	**1.263**	**0.116**	**3.24**	**-1.451**	**-8.645**	**-0.659**
C20:0	0.009	-0.261	0.114	-0.03	-0.112	0.185	**-0.302**	**-0.896**	**-0.006**
C22:6n-3	0.181	-1.048	0.593	**0.905**	**0.402**	**1.347**	-0.647	-5.007	0.237
C22:5n-3	0.164	-1.182	0.555	**0.749**	**0.547**	**1.833**	-1.508	-5.914	0.498
C22:0	0.027	-0.081	0.06	**0.053**	**0.026**	**0.144**	-0.016	-0.294	0.048
EPA + DHA	-0.281	-2.687	1.338	**2.006**	**0.564**	**4.621**	**-1.834**	**-13.628**	**-0.136**
EPA + DHA + DPA	0.099	-3.783	1.501	**2.765**	**1.425**	**6.254**	-3.488	-23.758	0.357
SFA	18.009	-111.898	21.973	-6.806	-36.818	48.051	-115.571	-287.41	24.38
MUFA	8.727	-124.707	36.206	-19.197	-64.078	64.313	-137.149	-324.607	33.141
PUFA	-2.593	-32.448	6.184	13.377	-1.081	32.037	**-31.311**	**-118.191**	**-1.849**
PUFA/SFA	0.002	-0.016	0.06	0.048	-0.034	0.081	-0.016	-0.023	0.264
n-3 PUFA	0.66	-6.219	2.108	**3.152**	**2.042**	**9.309**	-6.86	-32.513	0.965
n-6 PUFA	-3.034	-26.771	5.225	**9.832**	**2.582**	**22.603**	**-22.415**	**-81.32**	**-1.496**
n-6/n-3 PUFA	-0.074	-0.161	0.249	**-0.158**	**-0.454**	**-0.045**	0.109	-0.235	0.658

Est. (estimate of effect), Lower (lower 95% confidence interval), Upper (upper 95% confidence interval), Bold; P < 0.05

## Discussion

To our knowledge, this is the first study, as no previously published literature has described *SCD*, *FABP4*, and *FASN* lipogenic gene expressions in the ribeye muscles of supplemented TAW lambs. The processes of fatty acid metabolism in ruminants are complex. Unsaturated fatty acids (UFA) are converted into SFA due to microbial biohydrogenation in the rumen [23]. The increased levels of these SFA increase the risks of atherosclerosis and coronary heart disease in humans [24]. The modification of fat composition in meat and meat products by the inclusion of omega-3 LC-PUFA is, therefore, an excellent approach to the promotion and improvement of human health [25]. This study utilised a lamb finishing feeding trial with omega-3 fortified, conventional MSM whole grain and control diets to quantify the expression patterns of *SCD*, *FABP4*, and *FASN* genes in the ribeye muscle of TAW lambs.

### Stearoyl-CoA desaturase gene

The *SCD* gene enhances meat quality traits by modifying fat deposition and fatty acid composition [26]. It achieves this by catalysing the synthesis of cis-vaccenic acid from the conjugated linoleic acid (c9, t11 isomer) [27] and converting SFA to MUFA by inserting a double bond between carbon atoms Δ9 and Δ10 of C18:0 fatty acid to synthesise C18:1 c-9 fatty acid [22, 28]. Wang et al. [29] and Cedernaes et al. [30] described the expression of the *SCD* gene as an indicator of IMF development, hence, it could be an essential regulator of muscle metabolism as it aids lipid biosynthesis and depresses fatty acids degradation [31].

Dietary omega-3 oil fortification in this study did not lead to any significant change in the expression of the *SCD* gene in the ribeye muscle in agreement with previous studies that reported no change in *SCD* gene expression in Italian Simmental and Holstein bulls fed linseed [32] and Angus steers fed corn oil [33]. This was in contrast to other studies that reported down-regulation of *SCD* expression when soybean oil was substituted with 2.7% of linseed oil [34] in cattle and in lambs fed alfalfa [19]. Dietary omega-3 oils supplementation is reported to suppress the *SCD* gene expression through the retinoic acid receptor-mediated signaling pathway [35], hence the lack of difference observed herein is not clear and warrants further investigation. However, these results corroborate the lack of difference in total MUFA, and oleic acid observed in the muscle of these lambs between treatments [36].

### Fatty acid binding protein 4 gene

Lipid synthesis and growth in sheep are influenced by the action of the *FABP4* gene [37], and drives the transport, lipogenesis, lipolysis, absorption and storage of long chain fatty acids [38, 39] hence, it is a metabolic indicator of an animal’s capacity to store IMF [40] and contributes to regulating the tenderness of meat in ovine species [41]. It is also associated with the regulation of lipid metabolic syndrome, insulin resistance, diabetes and obesity [42, 43]. Transcription factors, including peroxisomal proliferator-activated receptor (PPAR) α, -β and -γ, are triggered by fatty acids or other hydrophobic ligands and are responsible for stimulating *FABP4* gene expression, which occurs mainly in the adipocytes [38, 39]. In the current study, *FABP4* gene expression was down-regulated in the ribeye muscle of TAW lambs given omega-3 diet in comparison to the MSM diet.

The expression did not differ between the control and the omega-3 diet in this study. Similarly, Vargas-Bello-Pérez et al. [44] reported no difference in *FABP4* expression in subcutaneous adipose tissue of dairy cows fed diet supplemented with fish oil compared to the unsupplemented cows. The lack of difference in *FABP4* expression between the control and omega-3 treatments in this study suggests that inclusion of omega-3 oils did not depress fat synthesis [44]. These findings explain the similar total fatty acids content observed in the ribeye muscle of the control and omega-3 treatments presented in our previous study [36]. However, more work is required to elucidate the impact of omega-3 oils fortification in feed on the *FABP4* gene expression in sheep.

In the current study, the *FABP4* gene was expressed more in the muscle of lambs on MSM than in those consuming the fortified omega-3 supplement, indicating that *FABP4* up-regulation is promoted by supplementation with MSM whole grains. Diets regulate the mechanisms governing IMF deposition by triggering transcription factors such as the PPARγ that influence the expression of *FABP4* gene that encode proteins involved in fat accumulation and differentiation of adipocytes in muscle tissues [45]. A study carried out by Yang et al. [46] on the influence of diets with varied levels of energy on the efficiency of fat deposition and fatty acid profiles of the yak muscle, revealed dense energy diets boosted the deposition and partial fatty acids content of this muscle primarily by up-regulation of mRNA expression of lipogenic genes including *FABP4*. However, the MSM whole grains diet in this study had similar energy composition with the omega-3 fortified diet (14.4 versus 15.1 MJ/Kg dry matter) indicating that the difference in *FABP4* expression was not influenced by the diet energy composition.

The higher *FABP4* gene expression in the MSM whole grain group and the lower expression in the omega-3 fortified group in this study were associated with higher fatty acid deposition levels in the omega-3 than in the MSM supplemented TAW lambs [36]. In addition, *FABP4* gene expression was positively correlated with EPA and DHA. Previous studies have also reported the association between *FABP4* gene expression and intramuscular fat content [40, 47] and backfat depth [48] in ruminants.

These results indicate that the *FABP4* gene has a significant function in the biosynthesis of fatty acids in the ribeye muscle of TAW lambs, and supplementary diet composition is an important factor influencing its expression.

### Fatty acid synthase gene

The *de novo* synthesis of LC fatty acids from acetyl-CoA and malonyl-CoA precursors is carried out by the *FASN* gene [49, 50]. Hence, the degree of *FASN* expression performs an important role in fat deposition [51]. Previous reports on *FASN* gene down-regulation in the *Longissimus dorsi* muscle of Italian Large White and Duroc pigs [52] and in cattle supplemented with corn oil have been reported [33]. These reports agree with our current results where the expression of *FASN* gene tended to be lower in the ribeye muscle of lambs consuming omega-3 diet. Previous studies suggest that dietary fat supplementation may reduce *FASN* gene expression by inhibiting the activity of sterol regulatory element-binding protein [53] and carbohydrate-responsive element-binding protein [54]. The *FASN* gene expression was negatively correlated with the n-6 PUFA, PUFA, MUFA, SFA, 20:0, 18:3n-3, 18:2n-6, 18:0 and 16:0. Dietary omega-3 supplementation is reported to enhance the level fatty acids composition in the plasma of cattle [55] and the muscle, liver, kidney and heart tissues in sheep [36]. The high fatty acids composition blocks the activation of the carbohydrate-responsive element-binding protein, consequently inhibiting the lipogenic genes expression and *de novo* lipogenesis [56].

## Conclusion

Dietary treatment influenced the composition of fatty acid and lipogenic gene expression in the ribeye muscle of supplemented TAW lambs. The *FABP4* gene was expressed 3 folds higher in the muscles of lambs fed MSM whole grain, but not in the omega-3 fortified diet. Furthermore, the *FABP4* gene expression was positively correlated with DHA, EPA, and PUFA/SFA ratio. There was a tendency for the *FASN* gene expression to be lower in the lambs fed omega-3 fortified diet compared to the control diet, and the expression was negatively correlated with most of the fatty acids. Diets had no substantial influence on *SCD* gene expression in this study and no correlation between expression and the fatty acids was detected. The findings herein buttress the point that dietary omega-3 may improve muscle n-3 PUFA through regulation of the *de novo* fatty acids synthesis in the ribeye muscle of TAW lambs.

## Methods

### Management of experimental animals

The reporting in the manuscript follows the recommendations of Kilkenny et al. [57] in the ARRIVE guidelines for animal research. The design of the experiment, animals and location are described previously [36]. In summary, the lamb finishing feeding trial was accomplished from April to June 2019 (Crown Agriculture’s commercial feedlot complex, Borenore, New South Wales, Australia). The feedlot complex was well-ventilated, equipped with automated feeding and watering systems and had a concrete floor spacing of 5 m^2^ per head. The feeding troughs were equipped with sensors for data capture of every individual lamb’s body weight, rumination time and feed intake which were automatically recorded and cloud-stored. The experimental animals comprised 75 six-months old TAW lambs with a mean liveweight of 30 ± 1.2 kg randomly allocated into three dietary treatments of twenty-five animals per group: (1) omega-3 oil-fortified grain pellets, (2) unfortified grain pellets (control) or (3) unfortified commercial MSM whole grain (MSM Stockfeeds, Manildra, NSW, Australia) pellets. Details of the nutrient compositions of these experimental diets have been published [36]. Furthermore, lambs had unlimited access to hay diet and water. This completely randomised study lasted 47 days including the initial 14-day adaptation period. At the conclusion of the feeding period, lambs were slaughtered humanely at Gundagai Meat Processing Plant according to Meat Standards Australia regulations.

Samples of the ribeye muscles were taken from the 12th and 13th ribs interface 24 h post-mortem and kept in a -80 °C freezer.

### RNA isolation, cDNA, and quantitative PCR

Total RNA was extracted from frozen ribeye muscle samples utilising the TRIzol™ Plus RNA Purification Kit (Invitrogen, Thermo Fisher Scientific, Victoria, Australia), and subsequently purified and DNase-treated with ezDNase™ Enzyme (Thermo Fisher Scientific, Victoria, Australia). Total RNA yield and quality were checked with a NanoDrop ND-1000 spectrophotometer (Thermo Fisher Scientific, Victoria, Australia) and QuantiFluor® RNA System (Promega, WI, USA). Complementary DNA was synthesized from 100 ng RNA using SuperScript™ IV VILO™ Master Mix Reverse Transcription Kit (Thermo Fisher Scientific, Victoria, Australia).

Twenty microliters quantitative polymerase chain reaction (qPCR) reactions were run in duplicate utilising the Fast SYBR Green Chemistry (Thermo Fisher Scientific, Victoria, Australia) with 250 nM primers and 6 µL template on a QuantStudio-3 Real-Time qPCR detection system (Applied Biosystem Inc.). This was carried out under fast-cycling settings of initial 50 °C for 2 min, then 95 °C for 2 min, then 50 cycles at 95 °C for 15s and finally 65 °C for 1 min.

### Primer design and housekeeping gene selection

The primers of target and housekeeping genes (Table 2) were designed in the Genious Program version 2.2 (http://www.geneious.com). The suitability of all primers was ascertained by employing a serial dilution of pooled cDNA to generate a standard curve. All primer pairs established acceptable efficiency (90–110%) and R-value (99%). Data normalization for the target FASN, FABP4 and SCD genes utilized two reference genes; the elongation factor 1 A (EF1A, formerly termed EF1α) and Peptidyl-prolyl cis-trans isomerase A (PPIA) using an expression ratio that was constant amongst all samples as the key selection criterion [58–60]. The values for the differential gene expression comparison were represented as fold-change values in Fig. 1, calculated using the ∆Ct metric.

**Table 2:** Primer sequences for target and reference genes

Gene	Primers		Annealing Temperature (Ta)	Amplicon (bp)
	Forward	Reverse		
FABP4	ATGAAAGAAGTGGGTGTGGGCTTT	TCCTGGCCCAATTTGAAGGACATC	65	149
FASN	CCACTTCCCACTGGAACAAGACAA	GGAGGCGTAATAGATGGTGCAGAG	65	166
SCD	AACACCCAGCTGTCAGAGAAAAGG	AACAGCAGGACACCAGGTTTGTAG	65	110
EF1A	CGTGAAAACCACCGTTAAACCTAA	TCGTGGTAGACTTCCCTGAATCTA	65	100
PPIA	TCACACGCCATAATGGTACTGGTG	TGGCAGTGCAAATGAAAAACTGGG	65	153

### Fatty acids analysis

Details of the fatty acid composition of ribeye muscle biopsy samples analysed by means of gas chromatography–mass spectrophotometry procedure was previously described by [36].

Briefly, total lipids in 1 g of un-homogenized muscle tissue samples were extracted overnight. The original phase was a single-phase overnight extraction utilizing CHCl3:MeOH: H2O (1:2:0.8 v/v). The second segment involved phase separation with the addition of CHCl3:Saline-Milli-Q H2O (1:1 v/v) followed by rotary evaporation of the lower chloroform phase at 40 °C to acquire total lipids. The extracted cumulative lipids were separated into lipid classes by thin-layer chromatography (TLC) using 100 mL of the lipid extract reconstituted in hexane. The extract was marked onto silica gel G plates (200 × 200 × 0.25 mm3) using a micropipette. The TLC plate was developed in an acetone/petroleum ether (1:3 v/ v) solvent system in a tank comprising a few crystals of butylated hydroxytoluene (BHT) to hinder oxidation. Triacylglycerols, cholesterol and free fatty acids migrated, while phospholipids remained at the origin of the plate. The phospholipids were scraped off the plate into clean screw-capped test tubes for transmethylation and eventual computation of the lipid conversion factor (LCF) of 0.912 based on fatty acids/g of total lipids (0.083 for phospholipids, 0.829 for triacylglycerols and 0% for cholesterol since cholesterol does not have any fatty acids). An aliquot from each total lipid extract was utilized for transmethylation with MeOH:CHCl3:HCl (10:1:1 v/v) for 2 h at 80 °C. Fatty acid methyl esters (FAME) were extracted thrice using hexane:CHCl3 (4:1 v/v). A known concentration of an internal standard (C19:0) was added in a 1500 µL vial encompassing the extracted FAME. The FAME was analyzed on a 7890B gas chromatograph (Agilent Technologies, Palo Alto, CA, USA) furnished with an EquityTM − 1 fused 15 m silica capillary column with 0.1 mm internal diameter and 0.1 μm film thickness (Supelco, Bellefonte, PA, USA), a flame ionization sensor, a split/ splitless injector and an Agilent Technologies 7683 B Series autosampler. The gas chromatograph settings were splitless mode injection; carrier gas He; original oven temperature 120 °C and then increased to 270 °C at flow rates of 10 °C / min and to 310 °C at 5 °C / min. The Agilent Technologies ChemStation software (Palo Alto, CA, USA) was used to measure fatty acid peaks. The fatty acid identities were established using a Finnigan Thermoquest GCQTM GC/MS fitted with an on-column injector and Thermoquest Xcalibur software (Austin, TX, USA). Fatty acid contents were calculated as follows: FA mg/100 g = (Total lipid) × (LCF [0.912]) × ([%FA]/100) × 1000, where 0.912 was the resultant lipid conversion factor.

### Statistical analysis

As gene expression data can exhibit variability and may not always follow a normal distribution, a non-parametric approach provides a suitable alternative for hypothesis testing. Therefore, in this study, data on gene expression, fat melting point (FMP), intramuscular fat (IMF), and fatty acids profiles of TAW MARGRA lamb were analysed using nonparametric statistics in R version 4.0.1. Kruskal-Wallis tests with Bonferroni’s adjusted p-values were used to test for differences in fold changes among dietary treatments. Relationships between variables were explored using Spearman correlation analysis. The effect of gene expression (fold change) on FMP, IMF, and fatty acids was investigated using a quantile regression model. Quantile regression, an extension of linear regression, is often preferred to linear regression because it is robust to outliers [61] and thus superior when the assumptions of linear regression are unmet. We initially fitted ***animal*** as a random effect, and the fixed effects of ***treatment*** (omega-3 oil-fortified grain pellets, control unfortified grain pellets and unfortified commercial MSM whole grain), ***lipogenic gene*** (FASN, FABP4 and SCD) and their ***interactions*** in the model. However, the fold changes in gene expression did not follow a normal distribution pattern, hence we used a non-parametric approach in our analysis instead of an individual-level model. Also, the samples emanated from lambs of the same age and breed, hence animal variability would have been at a minimum, if not negligible. Therefore, we used the quantile regression methodology instead of the classical linear regression, because the quantile regression analysis is robust to outliers and enables the estimation of all the conditional quantiles of the response variable instead of the mean as depicted below:

$${y}_{i}={x}_{i}^{T}{\beta }_{\theta }+{u}_{{\theta }_{i}} , {\text{Q}\text{u}\text{a}\text{n}\text{t}}_{\theta }\left({y}_{i}|{x}_{i}\right)={x}_{i}^{T}{\beta }_{\theta } ,$$

Where ${\text{Q}\text{u}\text{a}\text{n}\text{t}}_{\theta }\left({y}_{i}|{x}_{i}\right)$ denotes the $\theta -th$ conditional quantile of the response variable ${y}_{i}$, conditional on the set of covariates ${x}_{i}$. ${\beta }_{\theta }$ are the coefficients associated with each covariate for the $\theta -th$ quantile level.

The comparison between quantile regression and linear regression models is depicted in the Supplementary Fig. S1-S3. Alpha was set to 0.05 for all statistical comparisons.

### Electronic supplementary material

Below is the link to the electronic supplementary material.

Supplementary Material 1

## Data Availability

The datasets generated and/or analysed during the current study are not publicly available due to contractual confidentiality clause obligation, but are available from the corresponding author on reasonable request.

## List of Abbreviations

ALA: α-linolenic and
cDNA: Complementary deoxyribonucleic acid
DHA: Docosahexaenoic acid
EF1A: Elongation Factor 1-Alpha
EPA: Eicosapentaenoic acid
FABP4: Fatty acid binding protein 4
FASN: Fatty acid synthase
FMP: Fat melting point
IMF: Intramuscular fat
LA: Linoleic acids
MSM: Unfortified whole grain pellets
MUFA: Monounsaturated fatty acids
n-3 LC-PUFA: Omega-3 long-chain polyunsaturated fatty acid
n-6 PUFA: Omega-6 polyunsaturated fatty acids
PPAR: Peroxisomal proliferator-activated receptor
PPIA: Peptidyl-Prolyl Cis-Trans Isomerase A
qPCR: Quantitative polymerase chain reaction
RNA: Ribonucleic acid
SCD: Stearoyl-CoA desaturase
SFA: Total saturated fatty acids
TAW: Tattykeel Australian White
UFA: Unsaturated fatty acids
