# Supplementary material for: Differential expressions of FASN, SCD, and FABP4 genes in the ribeye muscle of omega-3 oil-supplemented Tattykeel Australian White lambs
Source: BMC Genomics. 2023 Nov 6;24:666. doi: 10.1186/s12864-023-09771-x (PMC10626737; doi:10.1186/s12864-023-09771-x)
Supplement: Supplementary file 1 — Supplementary Material 1 [file 12864_2023_9771_MOESM1_ESM.docx]

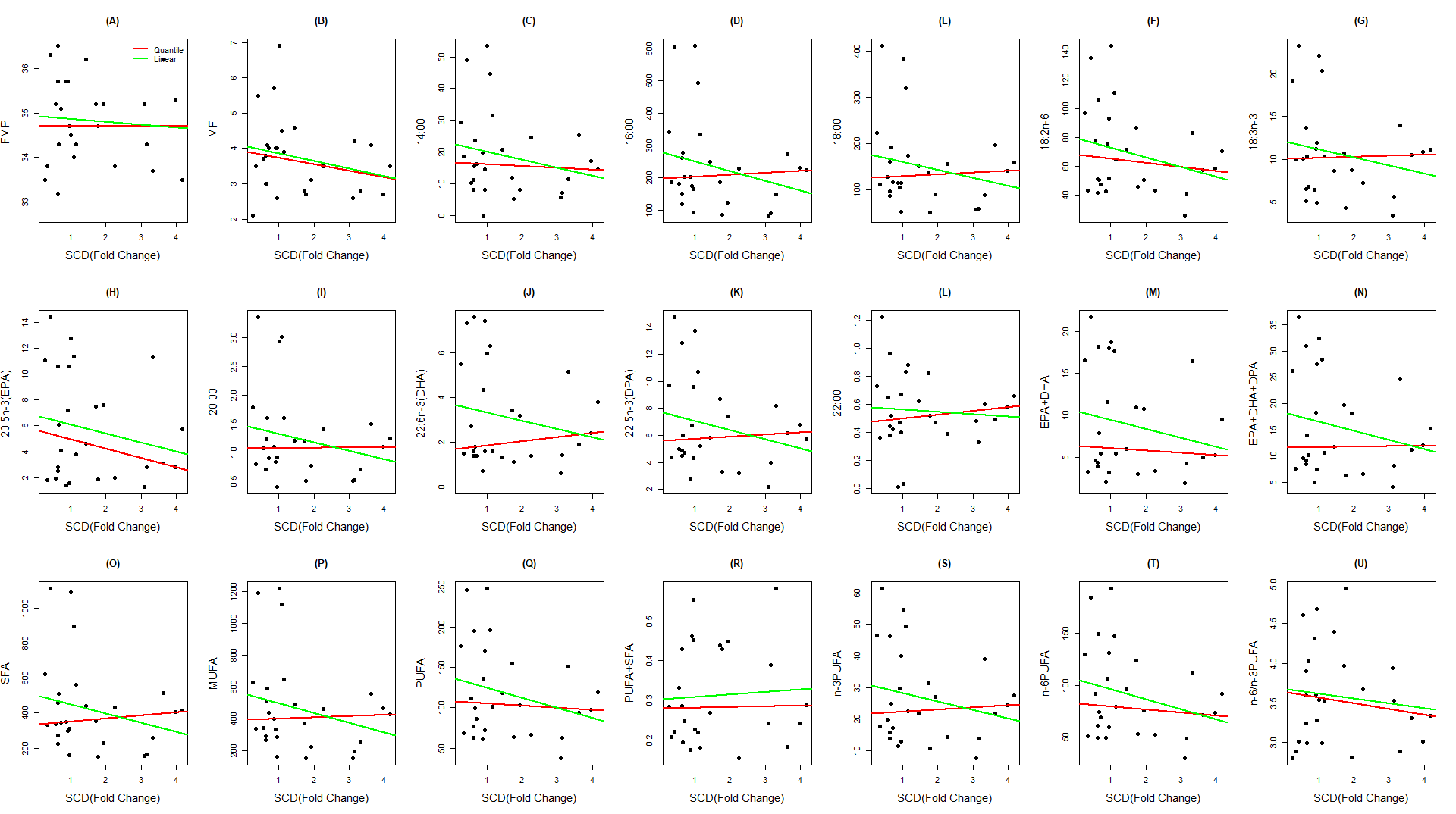


**Fig. S1** Scatterplot and regression fits (quantile and linear) for *SCD* expression (fold change) versus FMP, IMF and fatty acids. Red indicates fold changes, and green, meat quality traits.


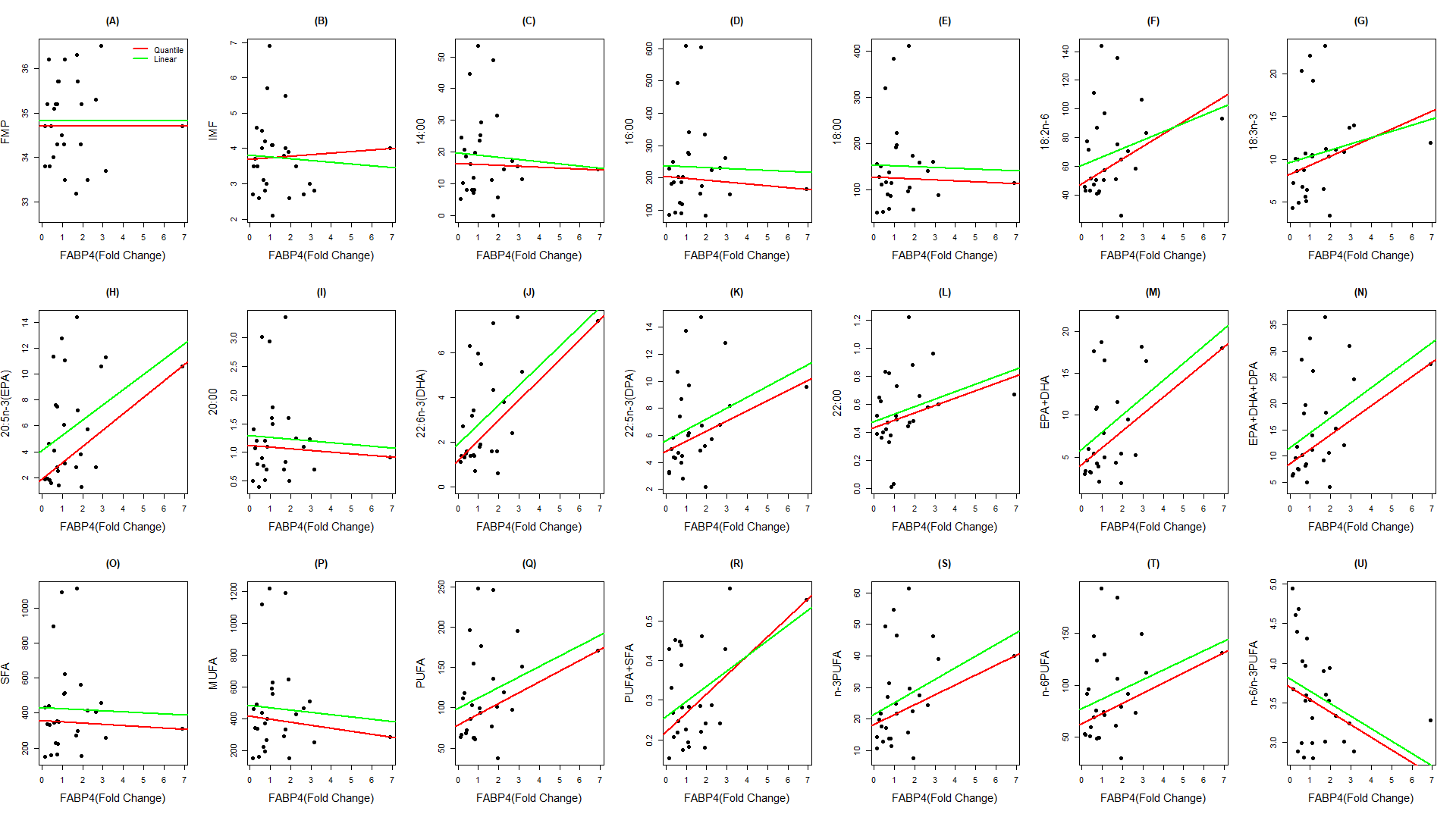


**Fig. S2** Scatterplots and regression fits (quantile and linear) for *FABP4* expression (fold change) versus FMP, IMP and fatty acids. Red indicates fold changes, and green, meat quality traits.


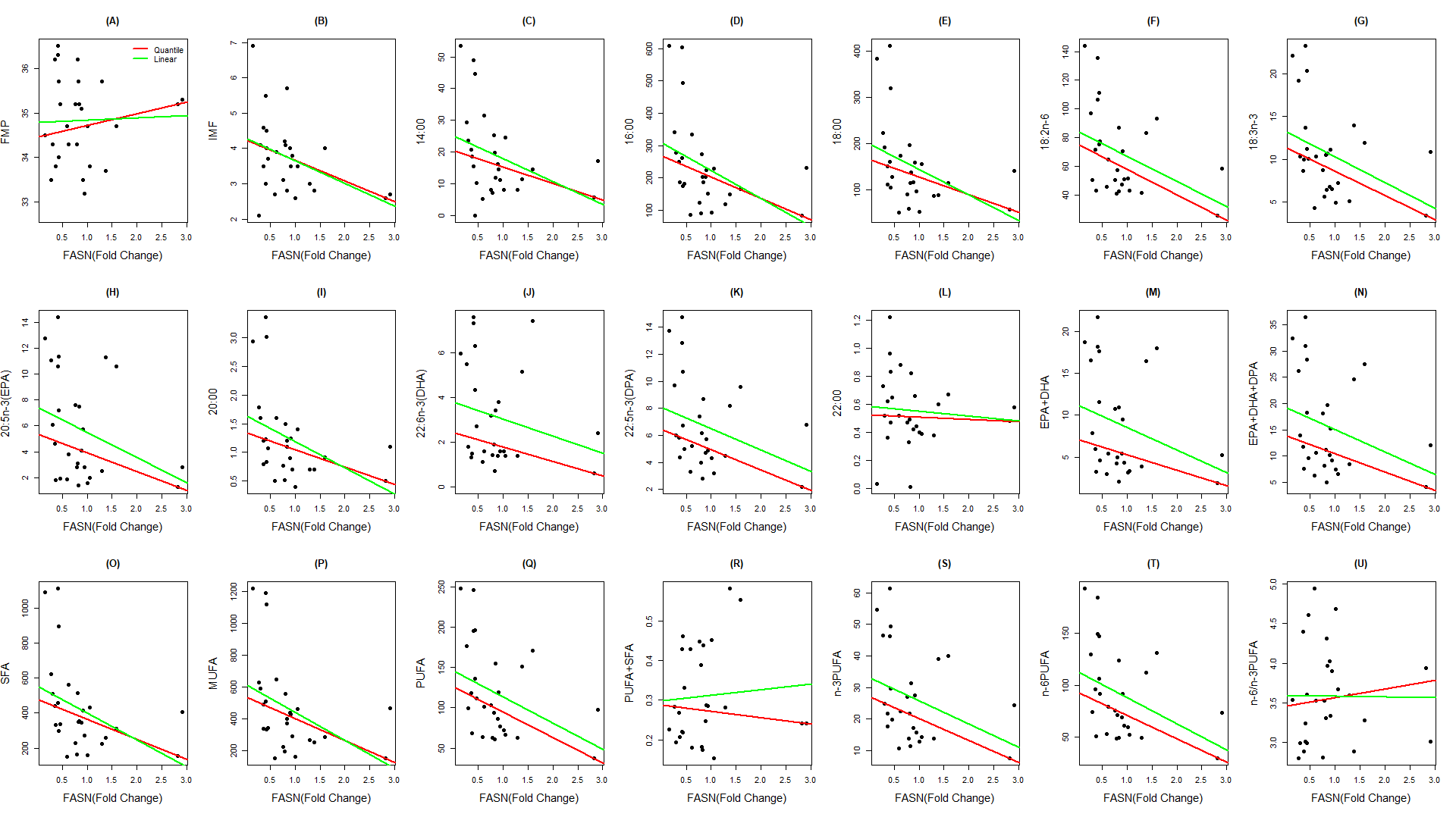


**Fig. S3** Scatterplots and regression fits (quantile and linear) for *FASN* expression (fold change) versus FMP, IMP and fatty acids. Red indicates fold changes, and green, meat quality traits.
